# Breast Cancer Classification from Ultrasound Images Using Probability-Based Optimal Deep Learning Feature Fusion

**DOI:** 10.3390/s22030807

**Published:** 2022-01-21

**Authors:** Kiran Jabeen, Muhammad Attique Khan, Majed Alhaisoni, Usman Tariq, Yu-Dong Zhang, Ameer Hamza, Artūras Mickus, Robertas Damaševičius

**Affiliations:** 1 Department of Computer Science, HITEC University Taxila, Taxila 47080, Pakistan; kiran.jabeen@student.hitecuni.edu.pk (K.J.); attique.khan@hitecuni.edu.pk (M.A.K.); 21-mscs-014@student.hitecuni.edu.pk (A.H.); 2 College of Computer Science and Engineering, University of Ha’il, Ha’il 55211, Saudi Arabia; m.alhaisoni@uoh.edu.sa; 3 College of Computer Engineering and Science, Prince Sattam Bin Abdulaziz University, Al-Kharaj 11942, Saudi Arabia; u.tariq@psau.edu.sa; 4 Department of Informatics, University of Leicester, Leicester LE1 7RH, UK; yudongzhang@ieee.org; 5 Department of Applied Informatics, Vytautas Magnus University, LT-44404 Kaunas, Lithuania; arturas.mickus@vdu.lt

**Keywords:** breast cancer, data augmentation, deep learning, feature optimization, classification

## Abstract

After lung cancer, breast cancer is the second leading cause of death in women. If breast cancer is detected early, mortality rates in women can be reduced. Because manual breast cancer diagnosis takes a long time, an automated system is required for early cancer detection. This paper proposes a new framework for breast cancer classification from ultrasound images that employs deep learning and the fusion of the best selected features. The proposed framework is divided into five major steps: (i) data augmentation is performed to increase the size of the original dataset for better learning of Convolutional Neural Network (CNN) models; (ii) a pre-trained DarkNet-53 model is considered and the output layer is modified based on the augmented dataset classes; (iii) the modified model is trained using transfer learning and features are extracted from the global average pooling layer; (iv) the best features are selected using two improved optimization algorithms known as reformed differential evaluation (RDE) and reformed gray wolf (RGW); and (v) the best selected features are fused using a new probability-based serial approach and classified using machine learning algorithms. The experiment was conducted on an augmented Breast Ultrasound Images (BUSI) dataset, and the best accuracy was 99.1%. When compared with recent techniques, the proposed framework outperforms them.

## 1. Introduction

Breast cancer is one of the most common cancers in women; it starts in the breast and spreads to other parts of the body [1]. This cancer affects the breast glands [2] and is the second most common tumor in the world, next to lung tumors [3]. Breast cancer cells create a tumor that might be seen in X-ray images. In 2020, approximately 1.8 million cancer cases were diagnosed, with breast cancer accounting for 30% of those cases [4]. There are two types of breast cancer: malignant and benign. Cells are classified based on their various characteristics. It is critical to detect breast cancer at an early stage in order to reduce the mortality rate [5].

Many imaging tools are available for the prior recognition and early treatment of breast cancer. Breast ultrasound is one of the most commonly used modalities in clinical practice for the diagnosis process [6,7]. Epithelial cells that border the terminal duct lobular unit are the source of the breast cancer. In situ or noninvasive cancer cells are those that remain inside the basement membrane of the draining duct and the basement membrane of the parts of the terminal duct lobular unit [8]. One of the most critical factors in predicting treatment decisions in breast cancer is the status of axillary lymph node metastases [9]. Ultrasound imaging is one of the most widely used test materials for detecting and categorizing breast disorders [10]. In addition to mammography, it is a common imaging modality used for performing radiological cancer diagnosis. The problems we may encounter in real life are not even reported. It is imperative to consider the presence of speckle, and to consider pre-processing such as wavelet-based denoising [11], in the first and second generations [12].

Ultrasound is non-invasive, well-tolerated by women, and radiation-free; therefore, it is a method that is frequently used in the diagnosis of breast tumors [9]. In dense breast tissue, ultrasound is a highly powerful diagnostic tool, often finding breast tumors that are missed by mammography [13]. Other types of medical imaging, such as magnetic resonance imaging (MRI) and mammography, are less portable and more costly than ultrasound imaging [14]. Computer-aided diagnosis (CAD) systems were developed to assist radiologists in the analysis of breast ultrasound tests [15,16]. Earlier CAD systems often relied on handmade visual information that is difficult to generalize across ultrasound images taken using different methods [17,18,19,20,21,22]. Recent developments have helped the construction of artificial intelligence (AI) technologies for the automated identification of breast tumors using ultrasound images [23,24,25]. A computerized method includes a few important steps such as the pre-processing of ultrasound images, tumor segmentation, extraction of features from the segmented tumor, and finally classification [26].

Recently, deep learning showed a huge improvement for cell segmentation [27], skin melanoma detection [28], hemorrhage detection [29], and a few more [30,31]. In medical imaging, deep learning was successful, especially for breast cancer [32], COVID-19 [33], Alzheimer’s disease recognition [34], brain tumor [35] diagnostics, and more [36,37,38]. CNN is a type of deep learning that includes several hierarchies of layers. Through CNN, image pixels are transformed into features. The features are later utilized for infection detection and classification. In CNN, the features are extracted from the raw images. The features extracted from the raw images also produce some irrelevant features that later affect the classification performance. Therefore, it is essential to select only the most relevant features for a better classification precision rate [39].

The selection of the best features from the originally extracted features is an active research topic. Many selection algorithms are introduced in the literature and applied in medical imaging, such as Genetic Algorithm (GA), Particle Swarm Optimization (PSO), and a few more. Using these methods, the best subset of the features instead of entire feature space. The main advantage of feature selection methods is that they improve system accuracy while decreasing computational time [40]. However, sometime during the best feature selection process, a few important features are also ignored, which impact on the system accuracy. Therefore, computer vision researchers introduced feature fusion techniques [41]. The fusion process increases the number of predictors and increases the accuracy of the system [42]. Some well-known feature fusion techniques are serial-based fusion and parallel fusion [43].

The following problems are considered in this article: (i) the available ultrasound images are not enough for the training of a good deep model as a model trained on a smaller number of images performs incorrect prediction; (ii) the similarity among benign and malignant breast cancer lesions is very high, which leads to misclassification; (iii) the features extracted from images contain irrelevant and redundant information that causes wrong predictions. To solve these problems, we propose a new fully automated deep learning-based method for breast cancer classification from ultrasound images.

The major contributions of this work are listed below.

- We modified a pre-trained deep model named DarkNet53 and trained it on augmented ultrasound images using transfer learning.
- The best features are selected using reformed deferential evolution (RDE) and reformed gray wolf (RGW) optimization algorithms.
- The best selected features are fused using a probability-based approach and classified using machine learning algorithms.

The rest of the manuscript is organized as follows. The related work of this manuscript is described in Section 2. Section 3 presents the proposed methodology, which includes deep learning, feature selection, and fusion. Results and analysis are discussed in Section 4. Finally, we conclude the proposed methodology in Section 5.

## 2. Related Work

Researchers present a number of computer vision-based automated methods for breast cancer classification using ultrasound images [44,45]. A few of them concentrated on the segmentation step, followed by feature extraction [46], and a few extracted features from raw images. Researchers used the preprocessing step in a few studies to improve the contrast of the input images and highlight the infected part for better feature extraction [47]. For example, Sadad et al. [48] presented a computer-aided diagnosis (CAD) method for the detection of breast cancer. They applied Hilbert Transform (HT) for reconstructing brightness-mode images from the rough data. After that, the tumor is segmented using a marker-controlled watershed transformation. In the subsequent step, shape, and textural features are extracted and classified using the K-Nearest Neighbor (KNN) classifier and the ensemble decision tree model. Badawy et al. [3] performed semantic segmentation, fuzzy logic, and deep learning for breast tumor segmentation and classification from ultrasound images. They used fuzzy logic in the preprocessing step and segmented the tumor using the semantic segmentation approach. Later, eight pre-trained models were applied for final tumor classification.

Mishra et al. [49] introduced a machine learning (ML) radiomics-based classification pipeline. The region of interest (ROI) was separated, and useful features were extracted. The extracted features were classified using machine learning classifiers for the final classification. The experimental process was conducted on the BUSI dataset and showed improved accuracy. Byra [14] introduced a deep learning-based framework for the classification of breast mass from ultrasound images. They used transfer learning (TL) and added deep representation scaling (DRS) layers between pre-trained CNN blocks to improve information flow. Only the parameters of the DRS layers were updated during network training to modify the pre-trained CNN to analyze breast mass classification from the input images. The results showed that the DRS method was significantly better compared with the recent techniques. Irfan et al. [5] introduced a Dilated Semantic Segmentation Network (Di-CNN) for the detection and classification of breast cancer. They considered a pre-trained DenseNet201 deep model and trained it using transfer learning that was later used for feature extraction. Additionally, they implemented a 24-layered CNN and parallel fused feature information with the pre-trained model and classified the nodules. The results showed that the fusion process improves the recognition accuracy.

Hussain et al. [50] presented a contextual level set method for segmentation of breast tumors. They designed a UNet-style encoder-decoder architecture network to learn high-level contextual aspects from semantic data. Xiangmin et al. [51] presented a deep doubly supervised transfer learning network for breast cancer classification. They introduced a Learning using Privileged Information (LUPI) paradigm, which was executed through the Maximum Mean Discrepancy (MMD) criterion. Later, they combined both techniques using a novel doubly supervised TL network (DDSTN) and achieved improved performance. Woo et al. [52] introduced a computerized diagnosis system for breast cancer classification using ultrasound images. They introduced an image fusion technique and combined it with image content representation and several CNN models. The experimental process was conducted on BUSI and private datasets and achieved notable performance. Byra et al. [53] presented a deep learning model for breast mass detection in ultrasound images. They considered the problem of variation in breast mass size, shape, and characteristics. To solve these issues, they performed selective kernel U-Net CNN. Based on this approach, they fused the information and performed an experimental process on 882 breast images. Additionally, they considered three more datasets and achieved improved accuracy.

Kadry et al. [54] created a computerized technique for detecting breast tumor section (BTS) from breast MRI slices This study employs a combined thresholding and segmentation approach to improve and extract the BTS from 2D MRI slices. To improve the BTS, a tri-level thresholding based on the Slime Mould Algorithm and Shannon’s Entropy is created, and Watershed Segmentation is implemented to mine the BTS. Following the extraction of the BTS, a comparison between the BTS and ground truth is carried out, and the required Image Performance Values are generated. Lahoura et al. [55] used an Extreme Learning Machine (ELM) to diagnose breast cancer. Second, the gain ratio feature selection approach is used to exclude unimportant features. Finally, a cloud computing-based method for remote breast cancer diagnostics is presented and validated on the Wisconsin Diagnostic Breast Cancer dataset.

Maqsood et al. [56] offered a brain tumor diagnosis technique based on edge detection and the U-NET model. The suggested tumor segmentation system is based on image enhancement, edge detection, and classification using fuzzy logic. The contrast enhancement approach is used to pre-process the input pictures, and a fuzzy logic-based edge detection method is utilized to identify the edge in the source images, and dual tree-complex wavelet transform is employed at different scale levels. The decaying sub-band pictures are used to calculate the features, which are then classified using the U-NET CNN classification, which detects meningioma in brain images. Rajinikanth et al. [57] created an automated breast cancer diagnosis system utilizing breast thermal images. First, they captured images of various breast orientations. They then extracted healthy/DCIS image patches, processed the patches with image processing, used the Marine Predators Algorithm for feature extraction and feature optimization, and performed classification using the Decision Tree (DT) classifier, which achieved higher accuracy (>92%) when compared with other methods. In [58], the authors presented a novel layer connectivity based architecture for the low contrast nodules segmentation from ultrasound images. They employed dense connectivity and combined it with high-level coarse segmentation. Later, the dilated filter was applied to refine the nodule. Moreover, a class imbalance loss function is also proposed to improve the accuracy of the proposed architecture.

Based on the techniques mentioned above, we discovered that most researchers do not pay attention to the preprocessing step. Typically, researchers performed the segmentation step first, followed by the extraction of features. A few of them used feature fusion to improve their classification results. They did not, however, concentrate on the selection of optimal features. They also ignored computational time, which is now an important factor. In this paper, we proposed an optimal deep learning feature fusion framework for breast mass classification. A summary of a few of the latest techniques is given below Table 1.

## 3. Proposed Methodology

**Figure 1:**
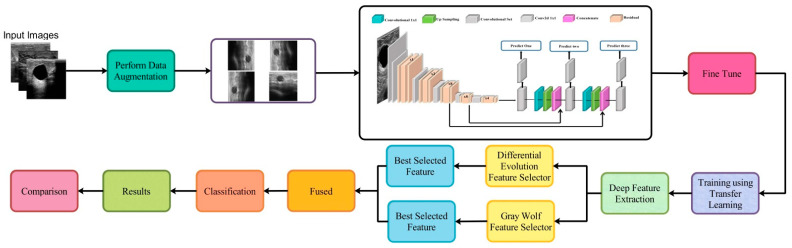
The proposed framework for breast cancer classification using Ultrasound Images.

The proposed framework for breast cancer classification using ultrasound images is presented in this section. Figure 1 illustrates the architecture of the proposed framework. Initial data augmentation is performed on the original ultrasound images and then passed to the fine-tuned deep network DarkNet53 for training purposes. Training is performed using TL and extract features from the global average pool layer. Extracted features are refined using the reformed feature optimization techniques, such as reformed differential evolution (RDE) and reformed gray wolf (RGW) algorithms. The best selected features are fused using a probability-based approach. Finally, the fused features are classified using machine learning classifiers. A detailed description of each step is given below.

### 3.1. Dataset Augmentation

Data augmentation has been an important research area in recent years in the domain of deep learning. In deep learning, neural networks required many training samples; however, existing data sets in the medical domain belong to the low resource domain. Therefore, a data augmentation step is necessary to increase the diversity of the original dataset.

**Figure 2:**
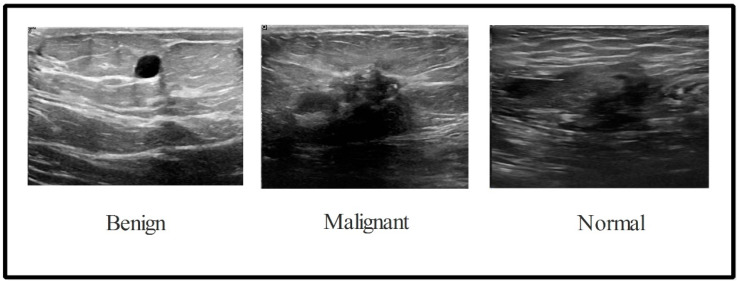
Sample ultrasound images of the BUSI dataset [59].

In this work, the BUSI dataset is used for the validation process. There are 780 images in the collection with an average image size of 500 × 500 pixels. This dataset consists of three total categories: normal (133 images), malignant (210 images), and benign (487 images) [59], as illustrated in Figure 2. We divided this entire dataset into the training and testing of ratio 50:50. After this, the training images of each class were normal (56 images), malignant (105 images), and benign (243 images). This dataset is not enough to train the deep learning model; therefore, a data augmentation step is employed. Three operations such as horizontal flip, vertical flip, and rotate 90 are implemented and performed on original ultrasound images to increase the diversity of the original dataset. These implemented operations are performed multiple times until the number of images in each class has reached 4000. After the augmentation process, the number of images in the dataset is 12,000.

### 3.2. Modified DarkNet-53 Model

**Figure 3:**
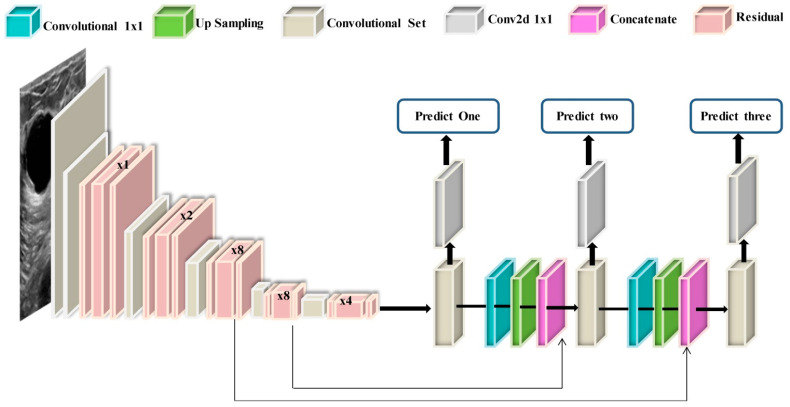
Structure of Modified DarkNet-53 deep model.

DarkNet-53 is a 53-layer deep convolutional neural network. It serves as the basis for the YOLOv3 object detection method. It can ensure super expression of features while avoiding the gradient problem produced by a too-deep network by combining Resnet’s qualities. The structure of the DarkNet-53 model is shown in Figure 3. It combines the residual network with the deep residual network. It contains successive 1×1 and 3×3 convolution layers and residual blocks. The convolutional layer is defined as follows:

(1) $$a_{m}^{n}=\underset{j\in X_{i}}{\sum }a_{j}^{n-1}*y_{jm}^{n}+z_{m}^{n}$$

In Equation (1), the input image is twisted by several convolution kernels to produce m separate feature maps $a_{m}^{n}$, which is represented in layer n by the m feature map. The symbol * represents the convolution operation. The feature vector of the image is represented by $X_{i}$ and the j element of the m convolution kernel in the layer n is represented by $y_{j}^{n}$.

The next important layer is the batch normalization (BN) layer.

(2) $$a_{out}=\frac{\propto \left(a_{m}^{n}-\partial \right)}{\sqrt{\omega^{2}+\varphi }}+\gamma$$

In Equation (2), the scaling factor is represented by ∝, the mean of all outputs is represented by ∂, the input variance is represented by ω, φ is a constant offset represented by γ, and the convolution calculation result is denoted by $a_{out}$. The result of BN denoted by $a_{out}$. The output is normalized using Batch Normalization corresponding to the same distribution of the coefficients of the same batch of eigenvalues. Following that, it has a convolutional layer that can accelerate network convergence, as well as avoiding over-fitting. The next layer is also known as an activation layer. In DarNet53, a leaky ReLu layer is included as an activation function. This function increases the nonlinearity of the network:

(3) $$x_{j}=\{\begin{array}{cc} y_{j}, & if\;a_{out}\geq 0\\ \frac{y_{j}}{b_{j}}, & if\;a_{out}<0 \end{array}$$

**Figure 4:**
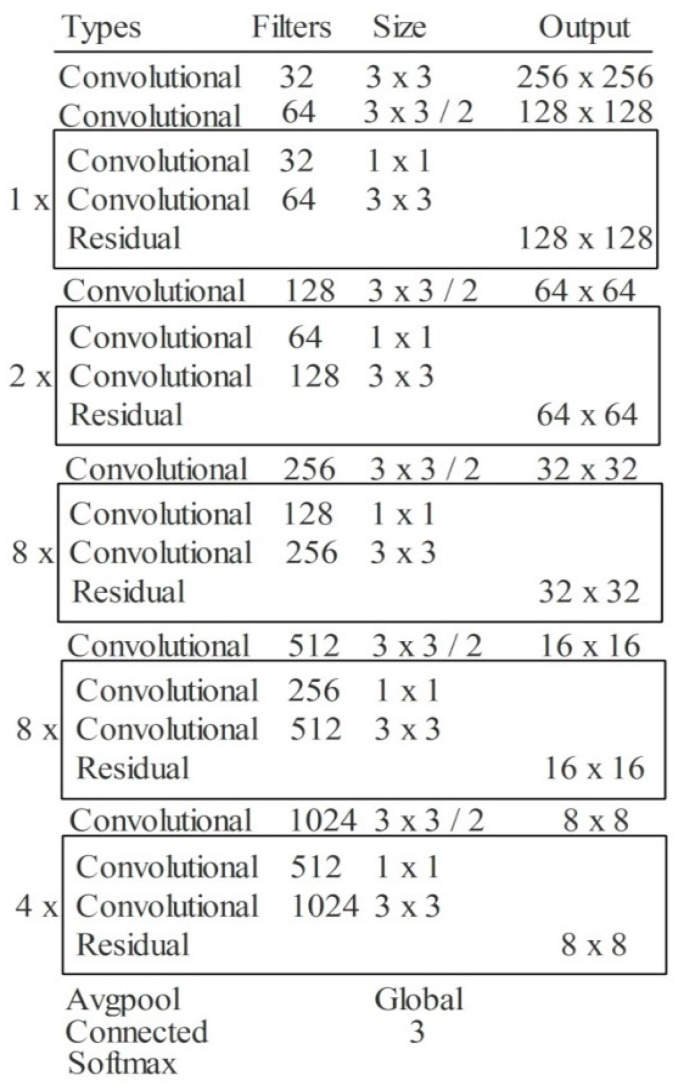
The layer wise architecture of Modified DarkNet-53 deep model.

In Equation (3), the input value is denoted by $y_{j}$, the activation value is represented by $x_{j}$, and the fixed parameter in the interval (1, +∞) is denoted by $b_{j}$. Another important layer in this network is pooling layer. This layer is employed for the downsampling of weights in the network. The max-pooling layer is used in this network. In the last example, all weights are combined in one layer in the form of a 1D array, also called features. These extracted features are finally classified in the output layer. The depth of this model is 53, the size is 155 MB, the number of parameters is 41.6 million, and the image input size is 256-by-256. The detailed layer-wise architecture is given in Figure 4.

### 3.3. Transfer Learning

Transfer learning (TL) is a machine learning approach in which a pre-trained model is reused for another task [60]. Reusing or transferring data from previous learned tasks for the newly learned tasks has the potential to dramatically improve the sampling efficiency of a supervised learning agent from a practical standpoint [61]. Here, TL is employed for the deep feature extraction. For this purpose, initially pre-trained model is fine-tuned and then trained using TL. Mathematically, TL is defined as follows:

A domain d={Y, p(y)} is described by two parameters: a feature space Y, and a distribution of marginal probabilities f(y), where y= {y1, y2, y3,…yn} ∈ Y. If there are two different domains, then they either have dissimilar marginal probabilities $\left(p\left(Y_{p}\right)\neq p\left(Y_{q}\right)\right)$ or feature space $\left(Y_{p}\neq Y_{q}\right)$.

Task: Given a particular domain d, there are two components of task t {X,g(.)}: a label space X, and a predictive function g (.); this is not visible, but can be derived from training data $\left\{(m_{j},n_{j}│j\left\{1,2,3,\ldots N\right\},\;\mathrm{where}\;m_{j}Y\;\mathrm{and}\;n_{j}\;X\right\}$. From a probabilistic point, $f\left(m_{j}\right)$ may also be written as $p\left(n_{j}|m_{j}\right)$, thus we can rewrite the task t as t={X, P(x|Y)}. If two tasks are dissimilar, their label spaces may differ $\left(X_{p}\neq X_{q}\right)$ or result in dissimilar distributions with conditional probabilities $\left(p\left(X_{p}|Y_{p}\right)\neq p\left(X_{q}|Y_{q}\right)\right)$.

**Figure 5:**
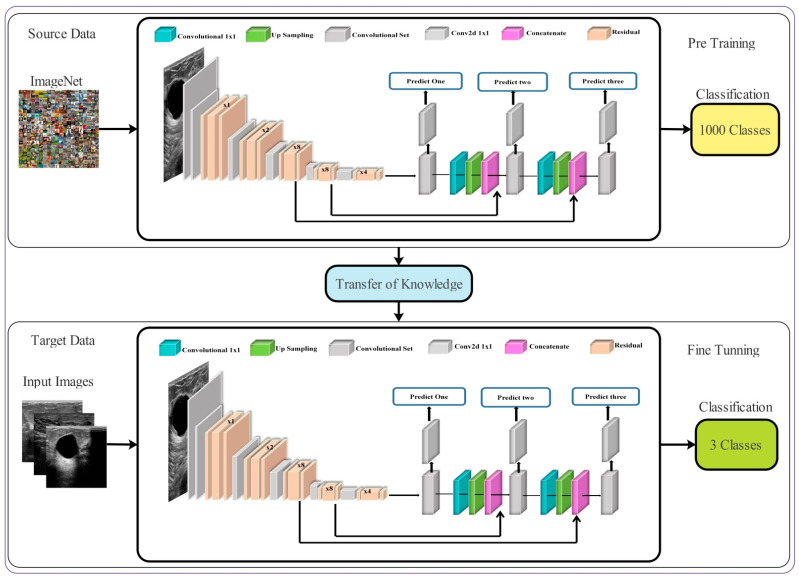
Transfer learning-based training of modified model and extract features.

The visual process of transfer learning is illustrated in Figure 5. The knowledge of the original model (source domain) is transferred to the modified deep model (target domain). After that, this modified model is trained, and the following hyper parameters are utilized: learning rate is 0.001, mini batch size is 16, epochs are 200, and the learning method is the stochastic gradient descent. The features are extracted from the Global Average Pooling (GAP) layer of the modified deep model. The extracted features are later optimized using two reformed optimization algorithms.

### 3.4. Best Features Selection

In this work, two optimization algorithms are reformed for the selection of best features such as differential evolution and gray wolf and fused their information for the final classification. The vector size after performing a differential evolution algorithm is 4788×818. Here, 818 is the number of features and 4788 is the number of images. The vector size after performing the gray wolf optimization algorithm is 4788×734.

#### 3.4.1. Reformed Differential Evolution (RDE) Algorithm

The DE algorithm searches the solution space using the differences between individuals as a guide. The DE’s main idea is to scale and differentiate two different specific vectors in the same population, then add a third individual vector to this population to generate a mutation independent vector, which is crossed with the parent independent vector with a certain possibility to produce an intended individual vector. Finally, greedy selection is applied to the generated individual vector and the parent independent vector, and the consistently better vector is preserved for the future generation. The DE’s fundamental evolution processes are as follows:

**Initialization:** D-dimensional vectors (D) are used as the starting solution in the DE algorithm. The population number can be represented by P, each independent factor can be denoted by $z_{j\;}\left(Y\right)=\left(z_{j1}\left(Y\right),\;z_{j2}\left(Y\right),z_{j3}\left(Y\right),\ldots ,z_{jn}\left(Y\right)\right)$, and $z_{j\;}\left(Y\right)\in$ denotes the deep extracted features. The starting population is produced in $\left[z_{min},z_{max}\right]$. Here, the number of D-dimensional vectors is denoted by D, population numbers are represented by P, and $z_{j}\left(Y\right)$ represents the jth individual.

(4) $$z_{jw}=z_{min}+rand\left(0,1\right)\times \left(z_{max}+z_{min}\right)$$

where Y denotes the Yth generation, the maximum and minimum values of the search space are representing by $z_{max}$ and $z_{min}$, respectively, and rand(0,1) indicates a random number that falls inside (0,1) the normal distribution.

**Mutation Operation:** The DE method generates a mutation vector $M_{j,Y}$ for each individual $z_{j,Y}$ in the existing population (target vector) using the mutation operation. A specific mutation technique can generate a relevant mutation vector for each derived target vector. Several DE mutation strategies are established based on the varied generating ways of mutation people. The five most widely utilized mutation techniques are:

DE/rand/1:

(5) $$M_{j,Y}=z_{r1,\;Y}+L\cdot \left(z_{r2,\;Y}-z_{r3,\;Y}\right)$$

DE/best/1:

(6) $$M_{j,Y}=z_{best,\;Y}+L\cdot \left(z_{r1,\;Y}-z_{r2,\;Y}\right)$$

DE/rand-to-best/1:

(7) $$M_{j,Y}=z_{j,\;Y}+L\cdot \left(z_{best,\;Y}-z_{j,\;Y}\right)+L\cdot \left(z_{r1,\;Y}-z_{r2,\;Y}\right)$$

DE/rand/2:

(8) $$M_{j,Y}=z_{r1,\;Y}+L\cdot \left(z_{r2,\;Y}-z_{r3,\;Y}\right)+L\cdot \;\left(z_{r4,\;Y}-z_{r5,\;Y}\right)$$

Random exclusive integers are created and denoted by $r_{1},\;r_{2},r_{3},r_{4}$ and $r_{5}$ within [1, D]. To scale a divergence vector, the scaling factor E is a positive constant value. In the Yth generation, $z_{best,\;Y}$ is an independent vector with the best global value.

**Crossover Operation:** To construct a test vector $v_{j,Y}=\left(v_{1,Y},v_{2,Y},v_{3,Y},\ldots ,v_{j,y}\right)$, each pair of target vectors $z_{j,Y}$ and their matching mutation vectors $M_{j,Y}$ are crossed.

A binomial crossover is defined as follows in the DE algorithm:

(9) $$v_{j,Y}=\{\begin{array}{ll} M_{j,Y} & if\;\left(rand_{i}\left(0,1\right)\leq C\right)\;or\;\left(i=i_{rand},\;i=1,2,3,\ldots ,K\right)\\ z_{j,y} & Otherwise \end{array}$$

where C denotes the crossover frequency and is a constant on [0,1]. This is used to limit the quantity of the duplicated mutation vector. The selected integer on [1,K], which is random, is denoted by $i_{rand}$.

**Selection operation:** If the parameter values reach the upper or lower bounds, they can be regenerated in a random and uniform manner within the specified range. The values of all the objective functions of the test vectors are then evaluated, and the selection operation is carried out. Each test vector’s objective function $f\left(v_{j,Y}\right)$ is matched to the associated target vector’s optimal solution value of the associated target vector in the current sample. If the test vector’s objective function is much less than or similar to the target vector’s, the target vector is replaced by the test vector for the upcoming generation. The target vector is kept for the following generation if this is not the case.

(10) $$z_{j,Y+1}=\{\begin{array}{ll} v_{j,Y} & if\;\left(f\left(v_{j,Y}\right)\leq f\left(z_{j,Y}\right)\right)\\ z_{j,Y} & Otherwise \end{array}$$

After obtaining the selected features $v_{j,Y}$, features are further refined using another threshold function called the selected standard error of mean (SSEoM). Using this new threshold function, the Sl(k) features are selected as a final phase.

(11) $$Tr=\{\begin{array}{ll} Sl\left(k\right) & for\;v_{j,Y}\geq SM\\ Ignore, & Elsewhere \end{array}$$

where Tr is a threshold function and SM is the standard error mean.

#### 3.4.2. Reformed Binary Gray Wolf (RBGW) Optimization

The key update Equation for bGWO1 in this approach is provided as follows:

(12) $$l_{j}^{h+1}=crossover\;\left(l_{1},l_{2},l_{3}\right)$$

crossover(l,m,n) is an appropriate crossover between solutions l,m,n and $l_{1},l_{2},l_{3}$, which are binary vectors showing the effect of wolves moving towards alpha, beta, and delta grey wolves, in that order. $l_{1},l_{2},l_{3}$ can be computed by using the following Equation (13):

(13) $$l_{1}^{t}=\{\begin{array}{ll} 1 & if\;\left(l_{a}^{t}+bistep_{a}^{t}\right)\geq 1\\ 0 & Otherwise \end{array}$$

where position vector in dimension t is denoted by $l_{1}^{t}$ and binary step is represented by $bistep_{a}^{t}$ in dimension t. It can be computed by using Equation (15):

(14) $$bistep_{a}^{t}=\{\begin{array}{ll} 1 & if\;costep_{a}^{t}\geq rand\\ 0 & Otherwise \end{array}$$

where rand is an integer picked at random from a uniformly distributed ∈[0,1], and the continuous value of the size step is denoted by $costep_{a}^{t}$; this can be computed by the following Equation (15):

(15) $$costep_{a}^{t}=\frac{1}{1+e^{-10\left(X_{1}^{t}Y_{a}^{t}-0.5\right)}}$$

where $X_{1}^{t}$ and $D_{a}^{t}$ are computed through Equations (16) and (17) that were later employed for the threshold selection as follows:

(16) $$\vec{X}=2c\cdot \vec{r_{1}}-c$$

(17) $$\vec{D_{\alpha }}=\left|\vec{C_{1}}\cdot \vec{X_{a}}-\vec{X}\right|$$

(18) $$l_{2}^{t}=\{\begin{array}{ll} 1 & if\;\left(l_{b}^{t}+bistep_{b}^{t}\right)\geq 1\\ 0 & Otherwise \end{array}$$

where in Equation (16), $\vec{X}$ is the updated position of prey, $\vec{r_{1}}$ denotes the random distribution, and c is constantly reduced in the scope of (2,0). In Equation (18), $\vec{D_{\alpha }}$ represent the distances of prey from each gray wolf and $\vec{C_{1}}$ represent the coefficient variable. In Equation (18), the position vector in dimension t is denoted by $l_{2}^{t}$ and the binary step is represented by $bistep_{b}^{t}$ in dimension t. It can be computed by using the following Equation (19):

(19) $$bistep_{b}^{t}=\{\begin{array}{ll} 1 & if\;costep_{b}^{t}\geq rand\\ 0 & Otherwise \end{array}$$

where rand is an integer picked at random from an uniformly distributed ∈[0,1] and the continuous valued of size step is denoted by $costep_{b}^{t}$; this can be computed by the following Equation (20):

(20) $$costep_{b}^{t}=\frac{1}{1+e^{-10\left(X_{1}^{t}D_{b}^{t}-0.5\right)}}$$

where $D_{b}^{t}$ in dimension t can be computed by Equation (21).

(21) $$D_{b}^{t}=\left|\vec{C_{2}}\cdot \vec{X_{b}}-\vec{X}\right|$$

(22) $$l_{3}^{t}=\{\begin{array}{ll} 1 & if\;\left(l_{c}^{t}+bistep_{c}^{t}\right)\geq 1\\ 0 & Otherwise \end{array}$$

where the position vector in dimension t is denoted by $l_{3}^{t}$ and the binary step is represented by $bistep_{c}^{t}$ in dimension t. It can be computed by using the following Equation (23):

(23) $$bistep_{c}^{t}=\{\begin{array}{ll} 1 & if\;costep_{c}^{t}\geq rand\\ 0 & Otherwise \end{array}$$

where rand is an integer picked at random and uniformly distributed ∈[0,1], and the continuous value of the size step is denoted by $costep_{c}^{t}$; this can be computed by Equation (24).

(24) $$costep_{c}^{t}=\frac{1}{1+e^{-10\left(X_{1}^{t}Y_{c}^{t}-0.5\right)}}$$

where $Y_{c}^{t}$ in dimension t can be computed by Equation (25).

(25) $$\vec{Y_{c}}=\left|\vec{S_{3}}\cdot \vec{R_{c}}-\vec{R}\right|a=1,$$

A stochastic crossover process is used per dimension to crossover u, v, w solutions.

(26) $$l_{t}=\{\begin{array}{ll} u_{t} & if\;rand<\frac{1}{2}\\ v_{t} & \frac{1}{2}\leq rand<\frac{2}{5}\\ w_{t} & Otherwise \end{array}$$

Binary values are $u_{t},\;v_{t}$ and $w_{t}$. These are three parameters in dimension t. The output of the crossover is denoted by $l_{t}$ in dimension t.

The algorithm is summarized in Algorithm 1.
**Algorithm 1.** Reformed Features Optimization Algorithm**Input:**g the pack’s total number of grey wolves, Gth the number of optimization iterations. **Output:**
$l_{\alpha }—$Binary position of the grey wolf that is optimal, $f\left(l_{\alpha }\right)—$Best fitness value **Begin**

  1.Create a population of g wolves with random positions ∈[0,1]  2.Find a, b, c solutions that are based on fitness.  3.**While** Criteria for stopping not met **do****For each**$wolf_{j}\in pack$**do**Calculate $l_{1},l_{2},l_{3}$ using Equations (13), (18) and (22).$l_{j}^{h+1}$ ← crossover among $l_{1},l_{2},l_{3}$ using Equation (26).**end** IUpdate c, X, S. IIExamine the individual wolf positions. IIIUpdate a, b, c.
**End**

### 3.5. Feature Fusion and Classification

The best selected features from the RDE and RGW algorithms are finally fused in one feature vector for the final classification. For the fusion of selected deep features, a probability-based serial approach is adopted. In this approach, initially probability is computed for both selected vectors and only one feature is employed based on the high probability value. Based on the high probability value feature, a comparison is conducted and features are fused in one matrix. The main purpose of this comparison is to tackle the problem of redundant features of both vectors. The final fused features are next classified using machine learning algorithms for the final classification. The size of the vector is 4788×704 after fusion.

## 4. Experimental Results and Analysis

Experimental Setup: During the training of fine-tuned deep learning model, the following hyper parameters are employed, such as a learning rate of 0.001, mini batch size of 16, epochs at 200, the optimization method is Adam, and the feature activation function is sigmoid. Moreover, the multiclass cross entropy loss function is employed for the calculation of loss.

All experiments are performed on MATLAB2020b using a desktop computer Core i7 with 8GB of graphics card and 16GB RAM.

The following experiments have been performed to validate the proposed method:

(i) Classification using modified DarkNet53 features in training/testing ratio of 50:50;
(ii) Classification using modified DarkNet53 features in training/testing ratio 70:30;
(iii) Classification using modified DarkNet53 features in training/testing ratio 60:40;
(iv) Classification using DE based best feature selection on training/testing ratio 50:50;
(v) Classification using the Gray Wolf algorithm based best feature selection in training/testing ratio 50:50, and
(vi) Fusion of best selected features and classification using several classifiers, including the support vector machine (SVM), KNN, decision trees (DT), etc.

The results of the proposed method are discussed in this section in terms of tables and visual plots. Different training and testing ratios are considered for analysis, such as 70:30, 60:40, and 50:50. The cross-validation value is selected at 10 for all experiments.

### 4.1. Results

The results of the first experiment are given in Table 2. This table presented the best accuracy obtained of 99.3% for Cubic SVM. A few other parameters are also computed for this classifier, such as sensitivity rate, precision rate, F1 score, FNR, and time complexity, and their values are 99.2, 99.2, 99.2, 0.8%, and 20.69 (s), respectively. The Q-SVM and MGSVM obtained the second-best accuracy of 99.2%. The rest of the classifiers such as ESD, LSVM, ESKNN, FKNN, LD, CGSVM, and WKNN and their accuracy values are 98.9%, 98.9%, 98.7%, 98.7%, 98.6%, 98.2%, and 97.9%, respectively.

**Figure 6:**
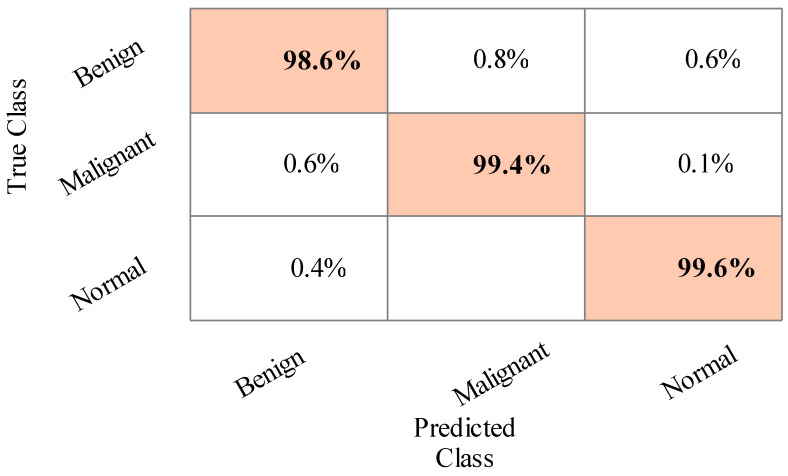
Confusion matrix of Cubic SVM for training/testing ratio of 50:50.

The sensitivity rate of Cubic SVM is validated through the confusion matrix illustrated in Figure 6. In addition, the computational time of each classifier is noted, and the best time is 120.909 (s) for LDA, and the worst time is 207.879 (s) for MGSVM.

**Figure 7:**
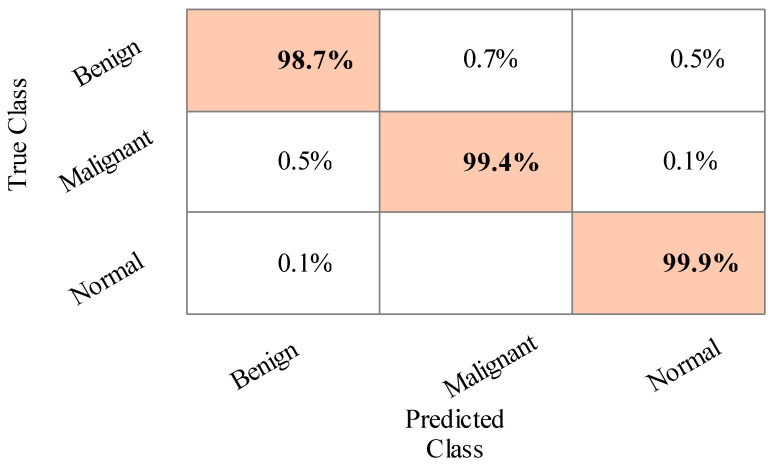
Confusion matrix of Cubic SVM for training/testing ratio of 70:30.

The results of the second experiment are given in Table 3. The best accuracy of 99.3% was obtained for Cubic SVM. A few other parameters are also computed, such as sensitivity rate, precision rate, F1 score, accuracy, FNR, and time complexity, and their values are 99.3%, 99.3%, 99.3%, 99.3%, 0.7%, and 11.112 (s), respectively. The MGSVM and Q-SVM classifiers obtained the second-best accuracy of 99.3% and 99.2%, respectively. The rest of the classifiers also achieved better performance. The confusion matrix of the Cubic SVM is illustrated in Figure 7. In addition, the computational time of each classifier is noted, and the minimum time is 111.112 (s) for the Cubic SVM, whereas the highest time is 167.126 (s) for ESD. When comparing the results of this experiment in Table 2, the classification accuracy is found to be consistent, but the computational time is minimized.

**Figure 8:**
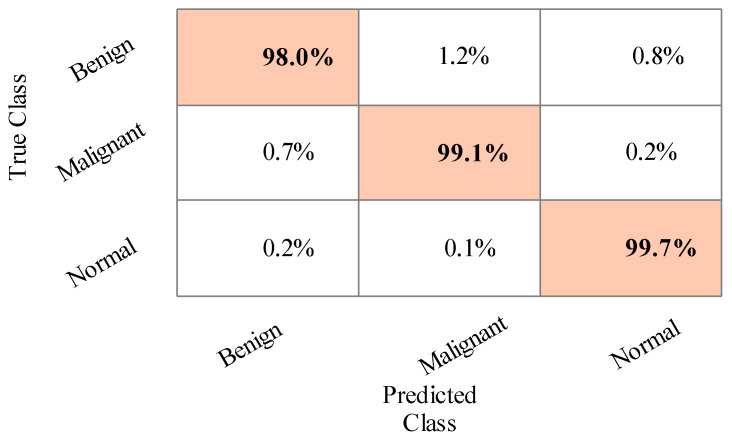
Confusion matrix of Cubic SVM for the training/testing ratio of 60:40.

**Figure 9:**
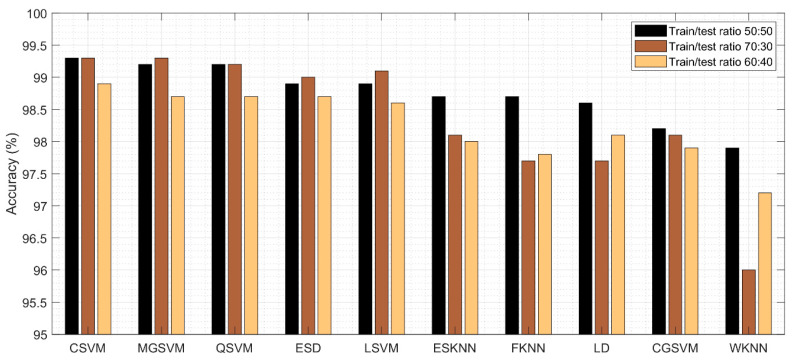
Summary of DarkNet53 classification accuracy using different training/testing ratios.

The results of the third experiment are given in Table 4. This table presented the best accuracy obtained at 98.9% for Cubic SVM. The MGSVM and Q-SVM obtained the second-best accuracy of 98.7%. The rest of the classifiers such as ESD, LSVM, ESKNN, FKNN, LD, CGSVM, and WKNN, and their accuracy values are 98.7%, 98.6%, 98%, 97.8%, 98.1%, 97.9% and 97.2%, respectively. The confusion matrix of Cubic SVM is illustrated in Figure 8. In addition, the computational time of each classifier is also noted, and the best time is 76.2 (s) for Cubic SVM and the worst time is 107.679 (s) for the ESKNN classifier. The accuracy of classifiers from experiments (i)–(iii) using different training/testing ratios is summarized in Figure 9. This figure illustrated that the performance at 50:50 is overall better than the rest of the selected ratios.

**Figure 10:**
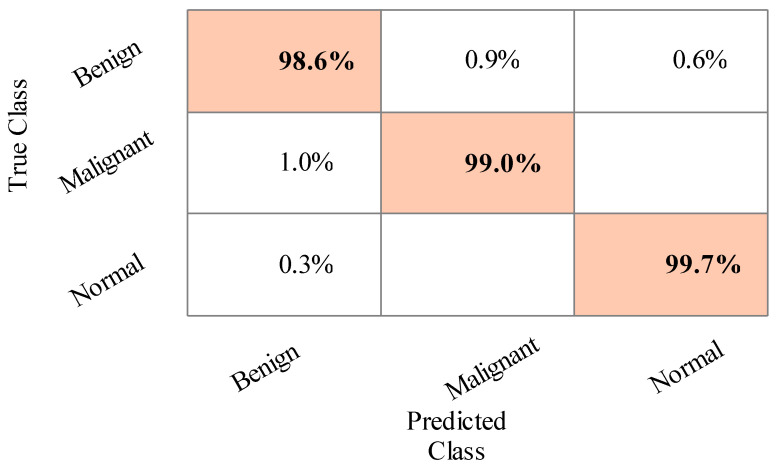
Confusion matrix of Cubic SVM for the features selected using DE and the train/test ratio of 50:50.

Table 5 presents the results of the fourth experiment. In this experiment, a 50:50 training/testing ratio is used. The best features are selected using the binary DE method. The 99.1% accuracy is achieved by Cubic SVM after feature selection. A few other parameters are also computed for this classifier, such as sensitivity rate, precision rate, F1 score accuracy, FNR, and time complexity, and their values are 99.1%, 99.06%, 99.08%, 1, 0.9 and 16.082, respectively. The confusion matrix of Cubic SVM is illustrated in Figure 10. The computational time of each classifier is also noted, and the best time is 28.082 (s) for the CSVM classifier, and the worst time is 42.829 (s) for the WKNN classifier. This shows that the computational time after the selection process is significantly minimized compared with the time given in Table 2 and Table 3.

**Figure 11:**
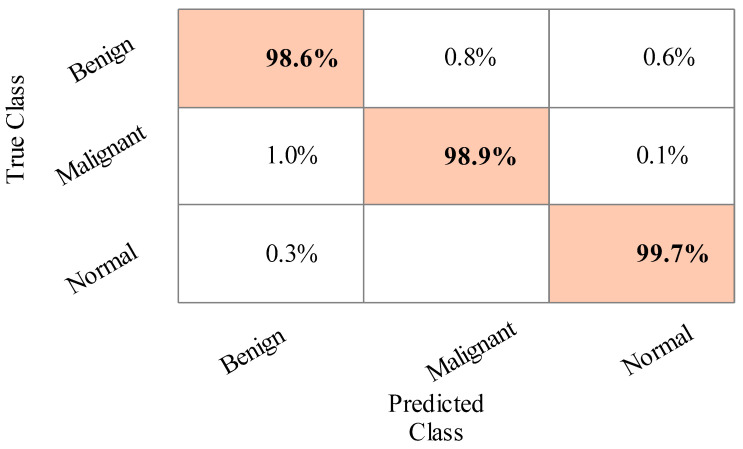
Confusion matrix of Cubic SVM for BGWO-based best feature selection.

The results of the fifth experiment are given in Table 6. In this experiment, the binary gray wolf optimization algorithm is implemented and selects the best features for the final classification. This table presents the best accuracy obtained of 99.1% for Cubic SVM. A few other parameters are also computed for this classifier, such as sensitivity rate, precision rate, F1 score, accuracy, FNR, and time complexity, and their values are 99.06%, 99.1%, 99.08%, 1, 0.94, and 15.239, respectively. The confusion matrix of Cubic SVM is illustrated in Figure 11. The computational time of each classifier is also noted, and the best time is 25.239 (s) for CSVM. This table shows that the overall time is minimized, and the accuracy is consistent with Table 2 and Table 3.

**Figure 12:**
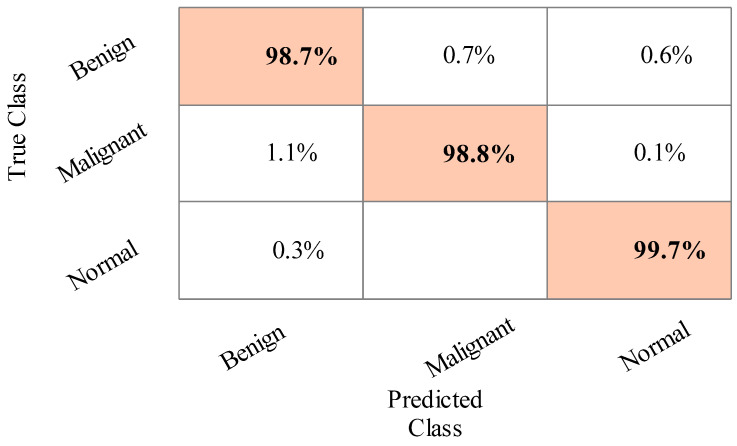
Confusion matrix of Cubic SVM after the proposed feature fusion approach.

Finally, the best selected features are fused using the proposed approach. The results are given in Table 7. This table presented the best accuracy obtained with 99.1% for Cubic SVM. The confusion matrix of Cubic SVM is illustrated in Figure 12. In this figure, the diagonal values show the correct predicted values. In addition, the computational time of each classifier is noted, and the best time is 13.599 (s) for the CSVM classifier.

**Figure 13:**
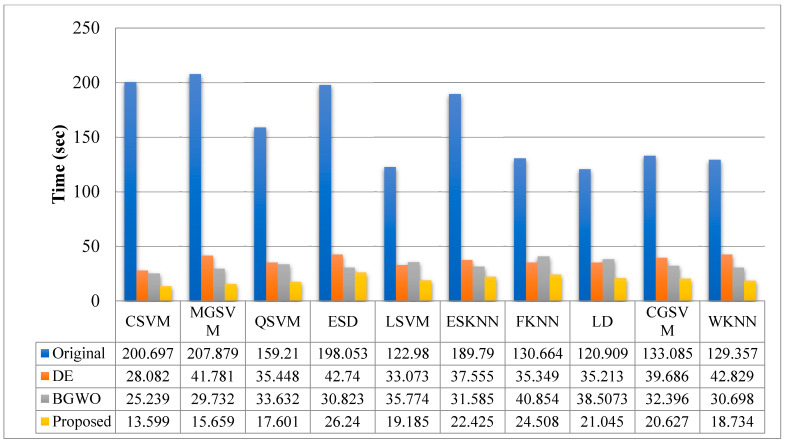
Computational time-based comparison of each step using the proposed framework.

Figure 13 compares the computational time while using the original features, the selected features based on DE, the feature selection based on BGWO, and feature fusion. In this figure, it is illustrated that the computational time of the original features is high, which was decreased after the feature selection step. Further, the proposed fusion process improves the performance in terms of computational time and consistency with the accuracy.

### 4.2. Statistical Analysis

For statistical analysis and comparison of the results, we used the post-hoc Nemenyi test. Demšar [62] has suggested using the Nemenyi test to compare techniques in a paired manner. The test determines a critical difference (CD) value for a given degree of confidence α. If the difference in the average ranks of two techniques exceeds the CD value, the null hypothesis, H0, that both methods perform equally well, is rejected.

**Figure 14:**
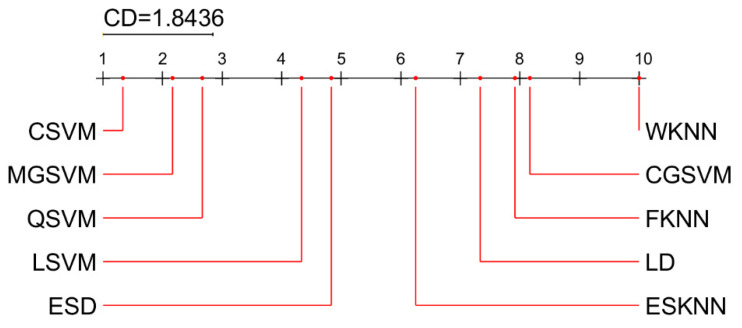
Critical difference diagram from the Nemenyi test: a comparison of classifiers (α = 0.05).

**Figure 15:**
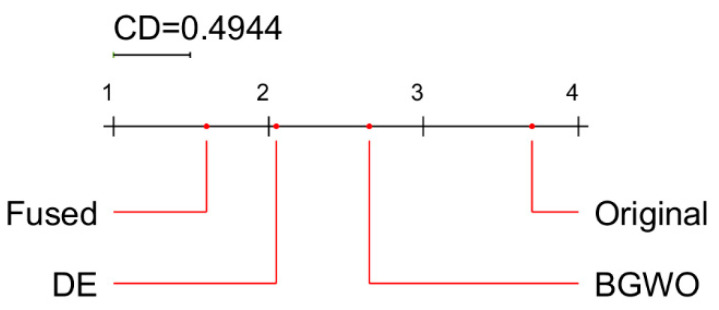
Critical difference diagram from the Nemenyi test: a comparison of feature selection methods (α = 0.05).

The results of statistical analysis are summarized in Figure 14 (mean ranks of classifiers) and Figure 15 (mean ranks of feature selection methods). The best classifier is CSVM, but MGSVM and QSVM also show very good results, in terms of accuracy, which are not significantly different from CSVM. The best feature selection method among the four methods analyzed is the proposed feature fusion approach, which is significantly better than other approaches (DE, BGWO, and original).

### 4.3. Comparison with the State of the Art

The proposed method is compared with the state-of-the-art techniques, as given in Table 8. In [63], the authors used ultrasound images and achieved an accuracy of 73%. In [64], the adaptive histogram equalization method was used to enhance ultrasound images and obtained an accuracy of 89.73%. In [52], a CAD system was presented for tumor identification that combines an imaging fusion method with various formats of image content and ensembles of multiple CNN architectures. The accuracy achieved for this data set was 94.62%. In [65], the source breast ultrasound image was first processed using bilateral filtering and fuzzy enhancement methods. The accuracy achieved was 95.48%. In [66], authors implemented a semi-supervised generative adversarial network (GAN) model and achieved an accuracy of 90.41%. The proposed method achieved an accuracy of 99.1% using a BUSi augmented dataset, where the computational time is 13.599 (s).

## 5. Conclusions

We proposed an automated system for breast cancer classification using ultrasound images. The proposed method is based on a few sequential steps. Initially, the breast ultrasound data are augmented and then retrained using a DarkNet-53 deep learning model. Next, the features were extracted from the pooling layer and then the best feature was selected using two different optimization algorithms such as the reformed BGWO and the reformed DE. The selected features are finally fused using a proposed approach that is later classified using machine learning algorithms. Several experiments were performed, and the proposed method achieved the best accuracy of 99.1% (using feature fusion and CSVM classifier). The comparison with recent techniques shows improvement in the results using the proposed framework. The strength of this work is: (i) augmentation of the dataset improved the training strength, (ii) the selection of best features reduced the irrelevant features, and (iii) the fusion method further reduced the computational time and consistency of accuracy.

In future, we will focus on two key steps: (i) increasing the size of the database, and (ii) designing a CNN model from scratch for breast tumor classification. We will discuss our proposed model with ultrasound imaging specialists and medical doctors, aiming for practical implementation at hospitals.

## Figures and Tables

**Table 1:** Summary of existing techniques for breast cancer classification.

Reference	Methods	Features	Dataset
[47], 2021	Shape Adaptive CNN	Deep learning	Breast Ultrasound Images (BUSI)
[48], 2020	Hilbert transform and Watershed	Textural features	BUSI
[3], 2021	Fuzzy Logic and Semantic Segmentation	Deep features	BUSI
[49], 2021	Machine learning and radiomics	Textural and geometric features	BUSI
[14], 2021	CNN and deep representation scaling	Deep features through scaling layers	BUSI
[50], 2020	U-Net Encoder-Decoder CNN architecture	High level contextual features	BUSI
[56], 2021	U-Net and Fuzzy logic	CNN features	BUSI

**Table 2:** Classification results of DarknNet53 using ultrasound images, where the training/testing ratio is 50:50.

Classifier	Sensitivity (%)	Precision (%)	F1 Score (%)	Accuracy (%)	FNR	Classification Time (s)
CSVM	99.2	99.2	99.2	99.3	0.8	200.697
MGSVM	99.2	99.2	99.2	99.2	0.8	207.879
QSVM	99.16	99.16	99.16	99.2	0.84	159.21
ESD	98.8	98.8	98.8	98.9	1.2	198.053
LSVM	98.93	98.93	98.93	98.9	1.07	122.98
ESKNN	98.6	98.6	98.6	98.7	1.4	189.79
FKNN	98.7	98.7	98.7	98.7	1.3	130.664
LD	98.6	98.6	98.6	98.6	1.4	120.909
CGSVM	98.16	98.2	98.17	98.2	1.84	133.085
WKNN	97.9	97.93	97.91	97.9	2.1	129.357

**Table 3:** Classification results of DarkNet53 using ultrasound images, where the training/testing ratio is 70:30.

Classifier	Sensitivity (%)	Precision (%)	F1 Score (%)	Accuracy (%)	FNR (%)	Classification Time (s)
CSVM	99.3	99.3	99.3	99.3	0.7	111.112
MGSVM	99.2	99.2	99.2	99.3	0.8	113.896
QSVM	99.16	99.2	99.17	99.2	0.84	125.304
ESD	99.0	99.03	99.01	99.0	1.0	167.126
LSVM	99.06	99.1	99.07	99.1	0.94	120.608
ESKNN	98.06	98.03	98.04	98.1	1.94	141.71
FKNN	97.73	97.76	97.74	97.7	2.27	124.324
LD	97.6	97.6	97.6	97.7	2.4	131.507
CGSVM	98.06	98.06	98.06	98.1	1.94	155.501
WKNN	96.03	96.13	96.07	96.0	3.97	127.675

**Table 4:** Classification results of DarkNet53 using ultrasound images, where the training/testing ratio is 60:40.

Classifier	Sensitivity (%)	Precision (%)	F1 Score (%)	Accuracy (%)	FNR (%)	Classification Time (s)
CSVM	98.9	98.9	98.9	98.9	1.1	107.697
MGSVM	98.7	98.7	98.7	98.7	1.3	103.149
QSVM	98.7	98.7	98.7	98.7	1.3	89.049
ESD	98.6	98.7	98.65	98.7	1.4	94.31
LSVM	98.5	98.5	98.5	98.6	1.5	68.827
ESKNN	97.9	98	97.95	98.0	2.1	79.34
FKNN	97.8	97.8	98.24	97.8	2.2	84.537
LD	98.1	98.1	98.1	98.1	1.9	85.317
CGSVM	97.8	97.9	98.34	97.9	2.2	76.2
WKNN	97.2	97.2	97.2	97.2	2.8	81.191

**Table 5:** Classification results of binary differential evolution selector using ultrasound images, where the training/testing ratio is 50:50.

Classifier	Sensitivity (%)	Precision (%)	F1 Score (%)	Accuracy (%)	FNR	Classification Time (s)
CSVM	99.10	99.06	99.08	99.1	0.9	28.082
MGSVM	99.13	99.13	99.13	99.1	0.87	41.781
QSVM	99.10	99.10	99.1	99.1	0.9	35.448
ESD	98.70	98.70	98.7	98.7	1.3	42.74
LSVM	98.90	98.86	98.88	98.9	1.1	33.073
ESKNN	98.40	98.36	98.38	98.4	1.6	37.555
FKNN	98.26	98.30	98.28	98.3	1.74	35.349
LD	98.50	98.50	98.5	98.5	1.5	35.213
CGSVM	98.43	98.43	98.43	98.4	1.57	39.686
WKNN	97.00	97.10	97.05	97.0	3.0	42.829

**Table 6:** Classification results of binary gray wolf optimization selector using ultrasound images, where the training/testing ratio is 50:50.

Classifier	Sensitivity (%)	Precision (%)	F1 Score (%)	Accuracy (%)	FNR	Classification Time (s)
CSVM	99.06	99.1	99.08	99.1	0.94	25.239
MGSVM	98.96	98.96	98.96	99.0	1.04	29.732
QSVM	98.96	98.96	98.96	99.0	1.04	33.632
ESD	98.5	98.5	98.5	98.5	1.5	30.823
LSVM	98.7	98.7	98.7	98.7	1.3	35.774
ESKNN	98.5	98.5	98.5	98.5	1.5	31.585
FKNN	98.36	98.36	98.36	98.4	1.64	40.854
LD	98.2	98.2	98.2	98.3	1.8	38.5073
CGSVM	98.06	98.1	98.08	98.1	1.94	32.396
WKNN	97.2	97.2	97.2	97.2	2.8	30.698

**Table 7:** Classification results using the feature fusion of DE and BGWO using ultrasound images, where the training/testing ratio is 50:50.

Classifier	Sensitivity (%)	Precision (%)	F1 Score (%)	Accuracy (%)	FNR (%)	Classification Time (s)
CSVM	99.06	99.06	99.06	99.18	0.94	13.599
MGSVM	99.10	99.10	99.10	99.16	0.9	15.659
QSVM	98.96	98.96	98.96	99.30	1.04	17.601
ESD	98.76	98.80	98.78	98.90	1.24	26.240
LSVM	98.93	98.90	98.91	99.00	1.07	19.185
ESKNN	98.56	98.60	98.58	98.90	1.44	22.425
FKNN	98.36	98.36	98.36	98.74	1.64	24.508
LD	98.40	98.40	98.40	98.40	1.60	21.045
CGSVM	98.20	98.20	98.20	98.30	1.80	20.627
WKNN	97.46	97.53	97.49	98.10	2.54	18.734

**Table 8:** Comparison with the state-of-the-art techniques.

Reference	Year	Accuracy (%)	Time (s)
Cao et al. [63]	2020	73.0	-
Illesnami et al. [64]	2021	89.73	-
Pang et al. [66]	2021	90.41	-
Moon et al. [52]	2020	94.62	-
Zhuang et al. [65]	2021	95.48	-
Proposed		99.1	13.599

## Data Availability

The dataset used in this paper is available from https://scholar.cu.edu.eg/?q=afahmy/pages/dataset (accessed on 20 November 2021).

## Abbreviations

CNN	Convolutional neural network
RDE	Reformed differential evaluation
RGW	Reformed differential evaluation
MRI	Magnetic resonance imaging
CAD	Computer-aided diagnosis
AI	Artificial intelligence
GA	Genetic algorithm
PSO	Particle swarm optimization
HT	Hilbert transform
KNN	K-Nearest neighbor
ML	Machine learning
ROI	Region of interest
TL	Transfer learning
DRS	Deep representation scaling
Di-CNN	Dilated semantic segmentation network
LUPI	Learning using privileged information
MMD	Maximum mean discrepancy
DDSTN	Doubly supervised TL network
BTS	Breast tumor section
ELM	Extreme learning machine
DT	Decision tree
SVM	Support vector machine
WKNN	Weighted KNN
QSVM	Quadratic SVM
CGSVM	Cubic gaussian SVM
LD	Linear discriminant
ESKNN	Ensemble subspace KNN
ESD	Ensemble subspace discriminant
FNR	False negative rate
